# Supplementary material for: pH-Responsible Doxorubicin-Loaded Fe3O4@CaCO3 Nanocomposites for Cancer Treatment
Source: Pharmaceutics. 2023 Feb 26;15(3):771. doi: 10.3390/pharmaceutics15030771 (PMC10053241; doi:10.3390/pharmaceutics15030771)
Supplement: Supplementary file 1 [file pharmaceutics-15-00771-s001.zip › pharmaceutics-2223010-supplementary.pdf]

*Supplementary Materials*

# pH-responsible Doxorubicin-loaded $Fe_3O_4$@$CaCO_3$ Nanocomposites for Cancer Treatment

Victoriya Popova, Yuliya Poletaeva, Alexey Chubarov and Elena Dmitrienko

Institute of Chemical Biology and Fundamental Medicine (ICBFM), Siberian Branch of Russian Academy of Sciences, 8 Lavrentiev Avenue, 630090 Novosibirsk, Russia

**Figure S1.** DLS size distribution of $Fe_3O_4$@$CaCO_3$ (0.45 mg/mL of $Fe_3O_4$ synthesis) by number (top), volume (middle) and intensity (bottom) obtained by adding 0.45 mg/mL. ........ 2

**Figure S2**. DLS size distribution of $Fe_3O_4$@$CaCO_3$ by number (top), volume (middle), intensity (bottom) after 5 months of storage at 7 °C in deionized water. The particle size was determined by DLS (139 ± 5 nm, PDI of 0.33 ± 0.01). ........ 3

**Figure S3.** The hydrodynamic diameter by DLS of $Fe_3O_4$@$CaCO_3$ in various solutions. ........ 3

**Figure S4.** Possible nanoparticle interactions with doxorubicin (DOX). ........ 4

**Figure S5.** The proportion of DOX release from $Fe_3O_4$@$CaCO_3$/DOX (capacity, 25–1900 µg/mg) at pH 4.0 (A, C); pH 6.0 (B, D). ........ 5

**Figure S6.** The confirmation of DOX-loading by photography. The left photography also shows magnetic behavior on the magnetic tube rack. ........ 5

**Figure S7.** Fluorescence and UV-vis spectra of $Fe_3O_4$@$CaCO_3$/DOX nanocomposites. The measurements weres carried out using 100 µl of the solution on the Clariostar (BMG Labtech, Ortenberg, Germany). ........ 5

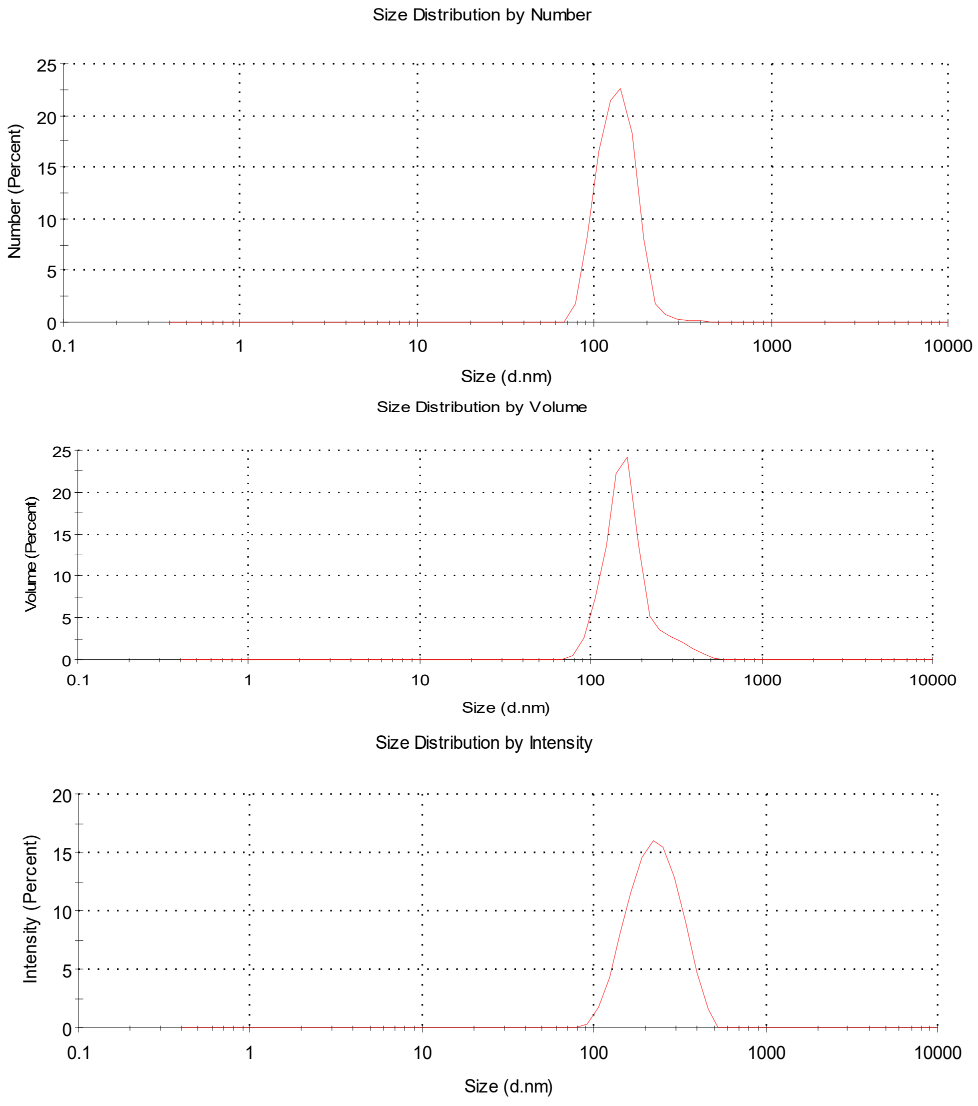


**Figure S1.** DLS size distribution of $Fe_3O_4$@$CaCO_3$ (0.45 mg/mL of $Fe_3O_4$ synthesis) by number (top), volume (middle) and intensity (bottom) obtained by adding 0.45 mg/mL.

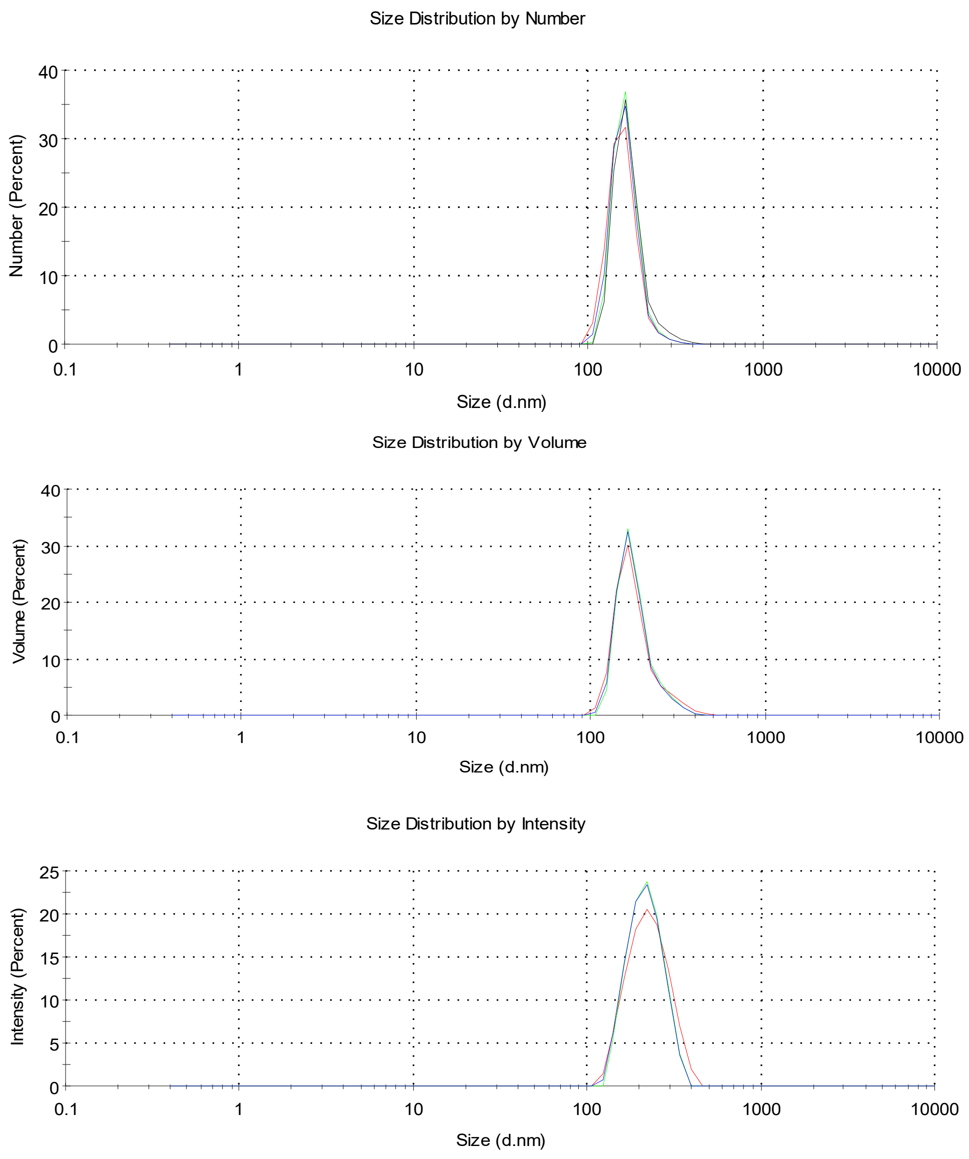


**Figure S2**. DLS size distribution of $Fe_3O_4$@$CaCO_3$ by number (top), volume (middle), intensity (bottom) after 5 months of storage at 7 °C in deionized water. The particle size was determined by DLS (139 ± 5 nm, PDI of 0.33 ± 0.01).

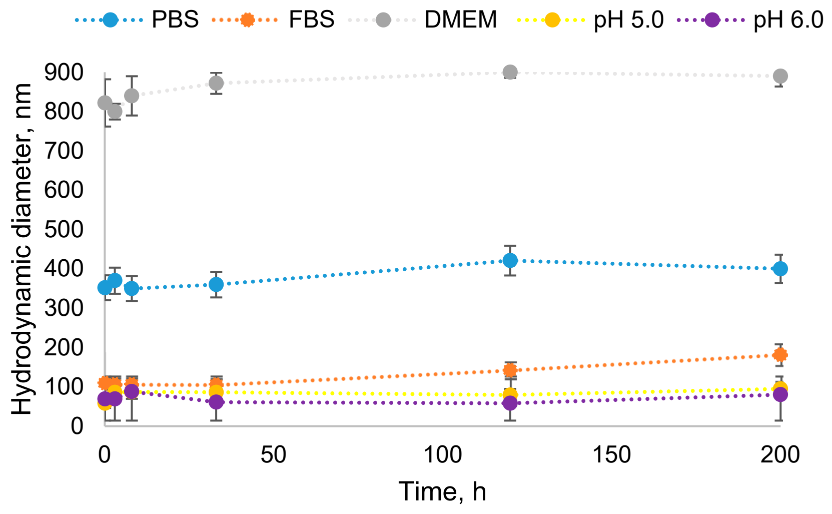


**Figure S3.** The hydrodynamic diameter by DLS of $Fe_3O_4$@$CaCO_3$ in various solutions.

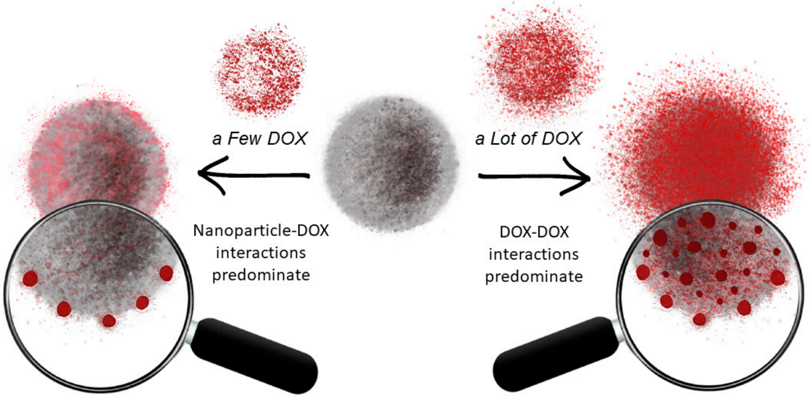


**Figure S4.** Possible nanoparticle interactions with doxorubicin (DOX).

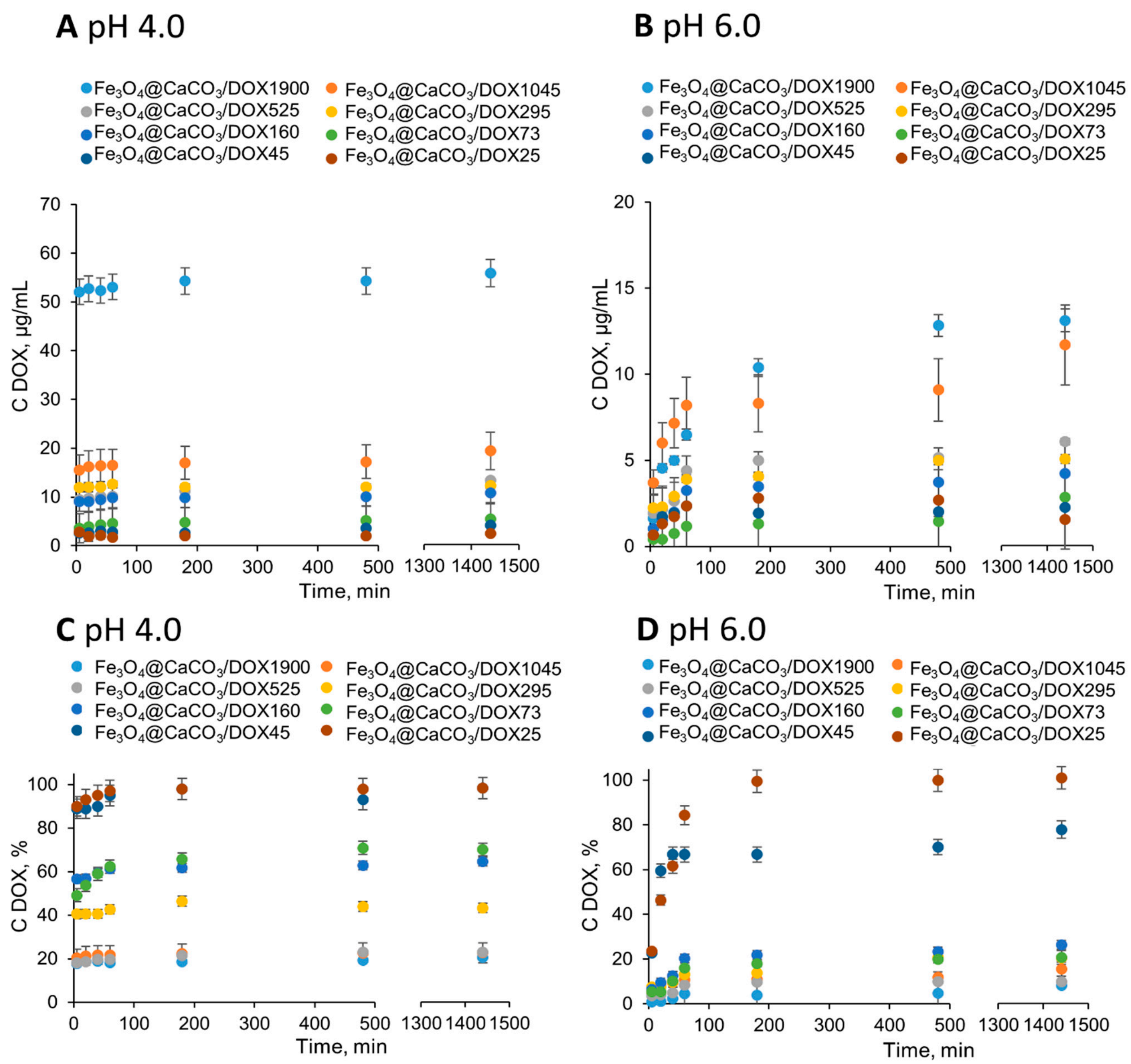

**Figure S5.** The proportion of DOX release from $Fe_3O_4$@$CaCO_3$/DOX (capacity, 25–1900 µg/mg) at pH 4.0 (A, C); pH 6.0 (B, D).

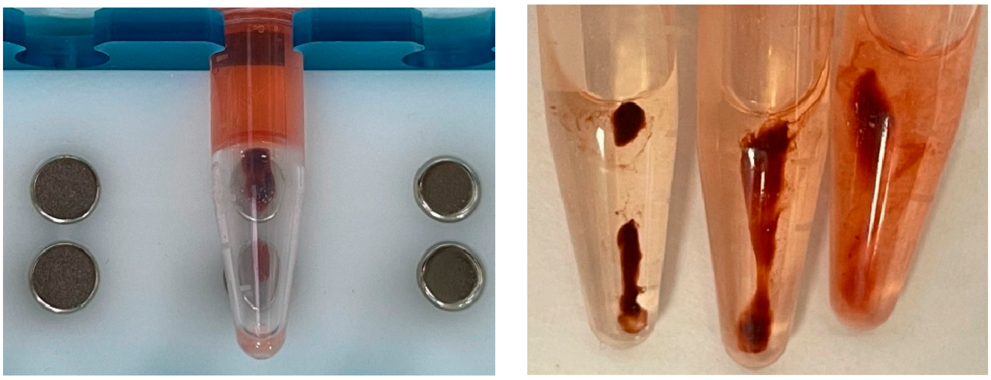

**Figure S6.** The confirmation of DOX-loading by photography. The left photography also shows magnetic behavior on the magnetic tube rack.

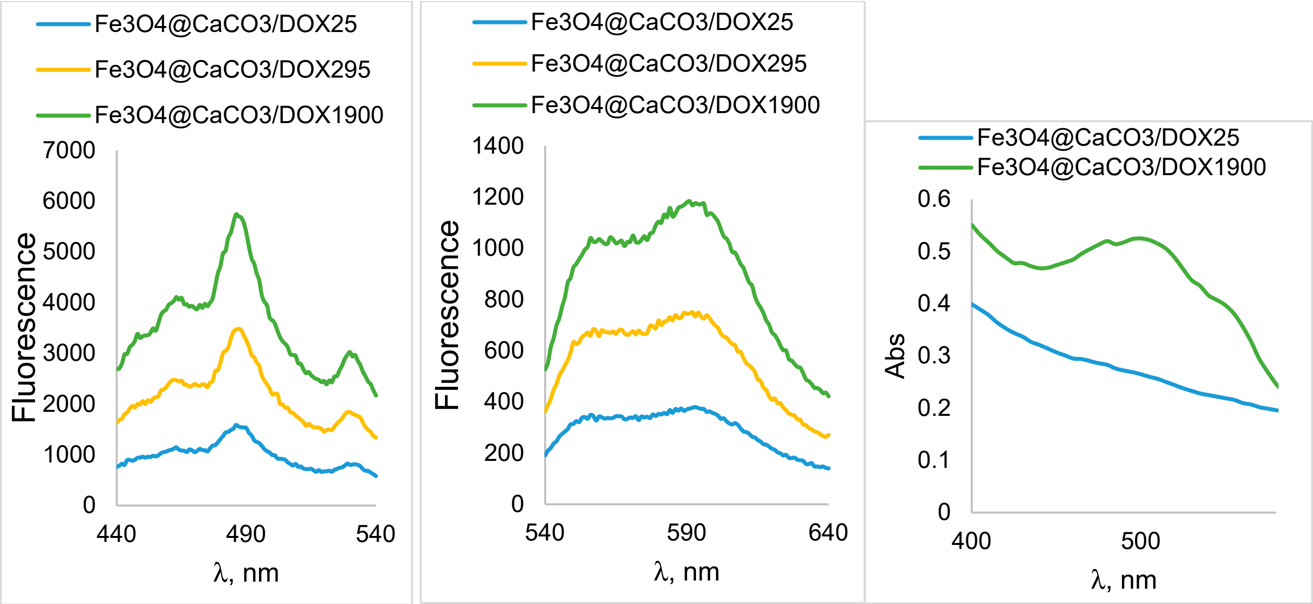


**Figure S7.** Fluorescence and UV-vis spectra of $Fe_3O_4$@$CaCO_3$/DOX nanocomposites. The measurements weres carried out using 100 µl of the solution on the Clariostar (BMG Labtech, Ortenberg, Germany).
